# The complete chloroplast genome sequence of *Origanum majorana* L

**DOI:** 10.1080/23802359.2020.1778561

**Published:** 2021-03-26

**Authors:** Le Thi Yen, Joonho Park

**Affiliations:** Department of Fine Chemistry, Seoul National University of Science and Technology, Seoul, South Korea

**Keywords:** Chloroplast genome, *Origanum majorana* L, Lamiaceae

## Abstract

Sweet marjoram (*Origanum majorana*) is an aromatic herb in the Lamiaceae family. This study aims to report the complete chloroplast nucleotide sequence of marjoram and the phylogenetic relationship with other Lamiaceae species. The total length of this plastome is 151,841 bp, containing a pair of inverted repeat regions (25,558 bp), separated by a large single copy region (83,035 bp) and a small single copy (17,690 bp). The genome encodes 132 genes, including 86 protein-coding genes, 36 tRNA genes, and eight rRNA genes. Phylogenetic tree analysis indicated that *O. majorana* is the most closely related to *Origanum vulgare*.

*Origanum majorana*, commonly known as sweet marjoram, is a creeping aromatic, perennial herb belonging to the genus *Origanum* L. in the mint family (Lamiaceae). Marjoram widely distributed in the Mediterranean region, North Africa, Europe, and western Asia and commonly used as a culinary additive to flavor many foods. *O. majorana* possesses strong pharmacological activities and Antioxidant activity due to its great diversity in essential oils, total phenolic compounds, and flavonoids (Vera 1999; Triantaphyllou et al. 2001; WenQi and Lei 2010). Therefore, this plant has been utilized as a folk medicine against rheumatism, gastrointestinal and respiratory problems, neurological disorders and headache (Bina and Rahimi 2017). Furthermore, the extraction of *O. majorana* has shown the protective effect against the aspartame-induced renal toxicity in female rats (Waggas et al. 2015), and the hypolipidemic effect of its mixture with chicory in obese rats (Ahmed et al. 2009), leaving a potential for the treatment of nephrotoxicity, obesity, and hyperlipidemia.

Herein, we aim to provide the complete chloroplast nucleotide sequence (cpDNA) of *O. majorana* and the phylogenetic relationship with other species in the family Lamiaceae. The fresh leaves of sweet marjoram were collected from Wanju-gun, Jeollabuk-do (35°52′49″N 127°11′23″E) and were deposited in the National Institute of Biological Resources, Incheon, Korea (NIBRVP0000773719). The total genomic DNA was extracted using CTAB method, and sequenced using the Illumina sequencing system (Illumina Inc., San Diego, CA). The annotation was conducted using GS Assembler version 7.

The complete chloroplast genome of *O. majorana* is 151,841 bp in length, consisting of one large single copy (LSC) of 83,035 bp, one small single copy (SSC) of 17,690 bp, and two inverted repeat (IRs) regions of 25,558 bp each. The overall GC content is 37.8%, with the highest content in IR (43%), followed by LSC (35.9%) and SSC (31.6%). A total of 132 genes is shown in the cpDNA, including 86 protein-coding genes, 38 tRNA genes, and eight rRNA genes. Among these genes, 18 genes are duplicated in IRs (*ndhB, rpl2, rpl23, rps7, rps12, rrn4.5, rrn5, rrn16, rrn23, trnA-UGC, trnI-CAU, trnI-GAU, trnL-CAA, trnN-GUU, trnR-ACG, trnV-GAV, ycf2, ycf15*). There are 18 intron-containing genes in the genome, with 16 genes containing a single intron (including 11 protein-coding genes and 5 tRNA genes) and two genes containing two introns each (*ycf3* and *clpP*). The complete chloroplast nucleotide sequence was deposited into GenBank with accession number

The phylogenetic construction from six Lamiaceae species was analyzed based on their cpDNA with following accession number: *Salvia chanryoenica* NC_040121, *Prunella vulgaris* NC_039654, *Mentha canadensis* NC_044082, *Thymus japonicus* NC_046822, *Origanum vulgare subsp. vulgare* JX880022, *Ocimum basilicum* NC_035143. Alignment of these whole plastomes was performed by using “build” function of ETE3 version 3.1.1 (Huerta-Cepas et al. 2016) and the maximum-likelihood tree was constructed using the FastTree version 2.1.8 with default parameters (Price et al. 2009). The reconstructed phylogeny indicated that *O. majorana* is the closest related to *O. vulgare* (Figure 1).

**Figure 1.**
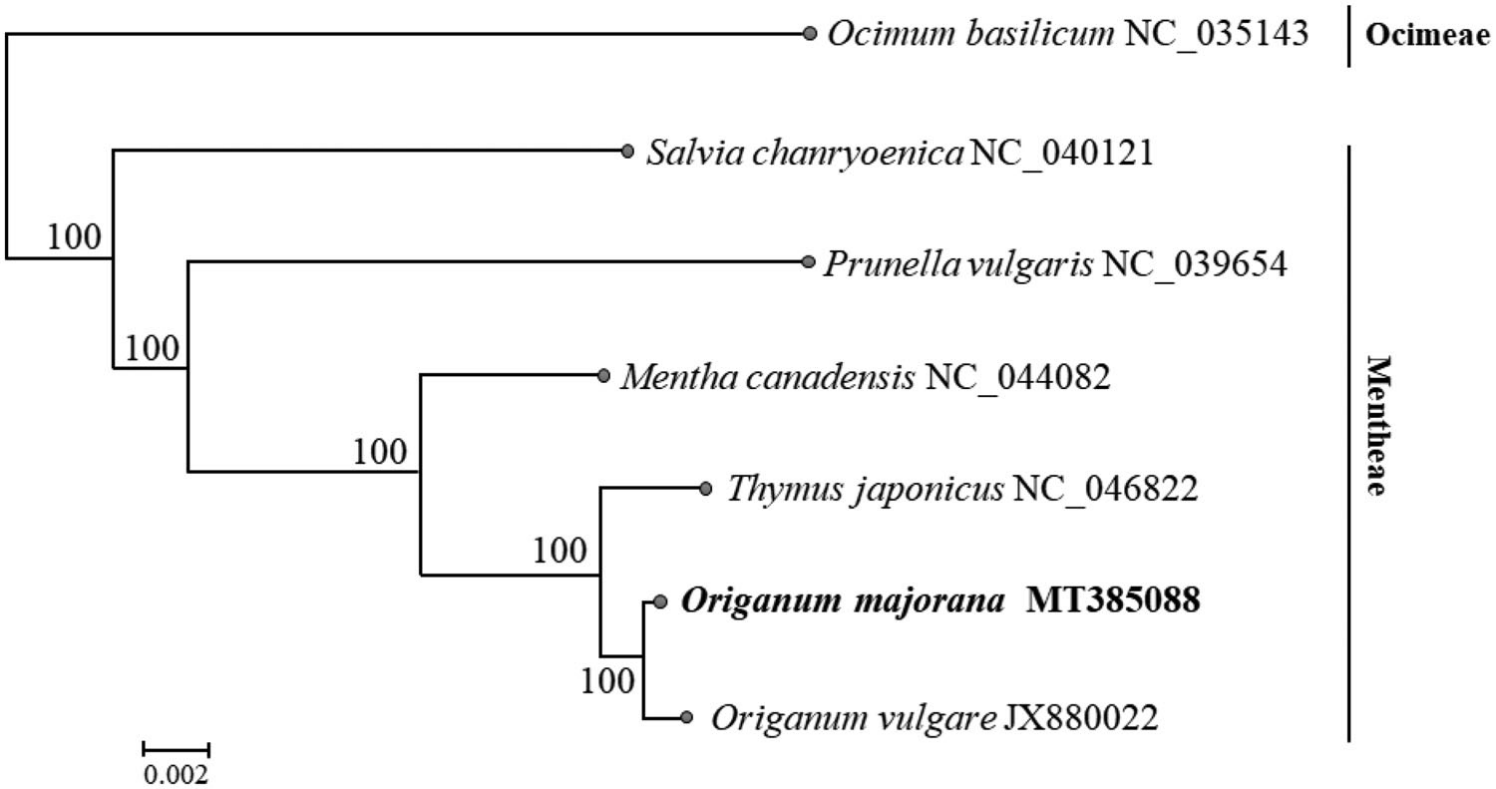
Phylogenetic tree constructed from complete genomes of 7 species using maximum-likelihood analysis with 1000 bootstrap replicates. Their accession number are as follows: *Ocimum basilicum* (NC_035143), *Salvia chanryoenica* (NC_040121), *Prunella vulgaris* (NC_039654), *Thymus japonicus* (NC_046822), *Origanum majorana* (MT385088), *Origanum vulgare* (JX880022).

## Data Availability

The data that support the findings of this study are openly available in GenBank of NCBI at https://www.ncbi.nlm.nih.gov, reference number MT385088.
